# De-novo malignancies after kidney transplantation: A long-term observational study

**DOI:** 10.1371/journal.pone.0242805

**Published:** 2020-11-30

**Authors:** Felix A. Fröhlich, Fabian Halleck, Lukas Lehner, Eva V. Schrezenmeier, Marcel Naik, Danilo Schmidt, Dmytro Khadzhynov, Katharina Kast, Klemens Budde, Oliver Staeck

**Affiliations:** Department of Nephrology and Medical Intensive Care, Charité Universitätsmedizin, Berlin, Germany; Imperial College Healthcare NHS Trust, UNITED KINGDOM

## Abstract

**Background:**

De-novo malignancies after kidney transplantation represent one major cause for mortality after transplantation. However, most of the studies are limited due to small sample size, short follow-up or lack of information about cancer specific mortality.

**Methods:**

This long-term retrospective analysis included all adult patients with complete follow-up that underwent kidney transplantation between 1995 and 2016 at our centre. All patients with diagnosis of malignancy excluding non-melanoma skin cancer (NMSC) were identified and a matched control group was assigned to the kidney transplant recipients with post-transplant malignancies.

**Results:**

1417 patients matched the inclusion criteria. 179 malignancies posttransplant were diagnosed in 154 patients (n = 21 with two, n = 2 patients with three different malignancies). Mean age at cancer diagnosis was 60.3±13.3 years. Overall incidence of de-novo malignancies except NMSC was 1% per year posttransplant. Renal cell carcinoma was the most common entity (n = 49, incidence 4.20 per 1000 patient years; cancer specific mortality 12%), followed by cancer of the gastro-intestinal tract (n = 30, 2.57; 50%), urinary system (n = 24, 2.06; 13%), respiratory system (n = 18, 1.54; 89%), female reproductive system (n = 15, 1.29; 13%), posttransplant lymphoproliferative disorders and haematological tumours (n = 14, 1.20; 21%), cancers of unknown primary (n = 7, 0.60 100%) and others (n = 22, 1.89; 27%). Male sex, re-transplantation and time on dialysis were associated with de-novo malignancies after transplantation.

**Conclusion:**

De-novo malignancies continue to be a serious problem after kidney transplantation. To improve long-term outcome after Kidney transplantation, prevention and cancer screening should be more tailored and intensified.

## Introduction

Transplantation is the best treatment for end-stage diseases of solid organs and provides increased patient survival, and better quality of life [1]. Besides the undisputable advantages of transplantation, an increased incidence of malignancies after solid-organ transplantation (SOT) was shown [2–5]. The risk varies for all malignancies and is believed to be multifactorial but possibly triggered by the chronic exposure to immunosuppressive agents [6–8]. With the improving long-term outcome after transplantation, de-novo malignancies have become one of the three major causes of death after transplantation while death from cardiovascular disease and infections are decreasing in frequency [9, 10].

Almost 2/3 of the transplanted organs worldwide in 2016 were kidneys and the kidney has been the most transplanted organ in Germany, too [11, 12]. With regard to this large number of kidney transplantations (KT), knowledge of malignancies after KT is increasingly important in all organ transplant programs. It was shown that de-novo malignancies after KT represent a serious problem and incidence has raised [13]. However, mortality might not be increased due to competing risk of death [14]. Calculating incidences and mortalities is difficult. Most of the epidemiological studies on posttransplant malignancies are limited due to small sample size, short follow-up intervals, lack of cancer specific mortality rates, or based on data from national registers. National registers have big advantages and cover a lot of patients but reporting of cancer to registries is often incomplete. As a consequence the true incidence and mortality might be underestimated. The present study systematically reviewed the medical records of the Universitätsmedizin Charité Berlin and data from the local cancer registry. It aims to describe risk factors, distribution, incidence and mortality of malignancies after KT.

## Materials and methods

All study procedures were approved by the institutional Ethics Committee (Ethikkommission Charité Berlin, Ethikausschuss 1 am Campus Charité Mitte; Antragsnummer: EA1/048/14).

### Inclusion criteria

The present study retrospectively investigated all adult patients (age at transplantation ≥ 18 years) with a complete follow-up, who underwent the first KT between 01.01.1995 and 31.12.2016 at our centre (n = 1417). The cohort was assembled using the clinical hospital documentation system and the documentation system of the department of transplantation. All patients were observed from the day of transplantation (the date of the first transplantation was chosen for re-transplanted patients) until 31/07/2017, the last visit in the centre or date of death, whichever occurred first. All participants signed a consent form and agreed to data acquisition for research studies when listed for transplantation. None of the transplant donors was from a vulnerable population and all donors or next of kin provided written informed consent that was freely given according to national laws and regulations within Eurotransplant. All medical costs, for organ donors and recipients were covered by the national health insurance systems. Living related and unrelated organ donation was according to German transplantation law. All living donors had undergone evaluation by an independent living donor ethics committee. Deceased donors were allocated through Eurotransplant regular allocation process.

### Immunosuppression

Most patients initially received a standard immunosuppressive protocol including induction therapy (anti-IL2-R antibody), calcineurin inhibitor, mycophenolate and steroids. ATG was rarely used in our cohort as initial immunosuppression [15]. Tapering of steroids was performed with the intention of achieving a steroid-free regimen after the first year, if no rejection episodes had occurred.

### Patient groups and malignancies

All included patients were screened for malignant tumours according to the ICD-10 classification. Non-melanoma skin cancer (NMSC; ICD-10 code: C44) was not assessed as malignancy in this study. Malignant tumours were identified using the electronic patient records of the outpatient clinic, the clinical information system and the database of the Charité Comprehensive Cancer Center (CCCC). The CCCC is a local cancer registry that integrates all information about tumour patients and also receives data from the residents´ local cancer registry (e.g. date of death). Consequently, the data about patients after KT at our centre is very complete and lacking hardly information. The time to the occurrence of the initial malignant cancer after KT was obtained for all individuals. The reoccurrence of the same cancer entity was counted as a single tumour event. For patients whose first malignancy appeared before the date of transplantation the time to the first occurrence of any subsequent cancer posttransplant was used in this analysis. If no malignant cancer posttransplant was diagnosed during the time of observation, patients were added to the control group.

Each tumour patient was assigned to one patient of the tumour free control group, the “matched control”. Consequently number of matched controls is identical to number of patients with malignancy. The goal of this matching procedure was to reduce bias due to epidemiological differences between the tumour free control group and the patients with tumour. Hence, the controls were matched in time of observation after KT (time under immunosuppression could not be shorter in matched controls), in patient´s age (age at transplantation ±4 years) and era of transplantation (year of transplantation ±4 years). If the malignancy was sex-related (e.g. cancer of the prostate gland) the sex had to be identical. If there was more than one possible match the same sex was preferred.

### Statistics

SPSS (Version 24.0.0.0, IBM, Chicago, IL, USA) was used to calculate all results. Results are displayed as average +/- standard deviation (SD) if skewness was between -1 and 1, otherwise mean +/- SD or IQR are shown. Data were tested for significant differences on the level p<0.05 either using the t-test (normally distributed data) or Man-Whitney-U-test (other data). The cumulative risk of malignancy, the predicted incidence of cancer, the survival after transplantation and the survival after the diagnosis of a malignancy were calculated by using the Kaplan-Meier-Method.

## Results

### Epidemiological results after renal transplantation

1417 Patients (39% females) matched the inclusion criteria of whom 154 developed 179 malignancies (23 patients had multiple malignancies) during the period of observation (11666 patient years). Previous analyses described time under immunosuppression (“time of observation”) and age at transplantation as two major predictors for de-novo malignancies after SOT that bias comparisons on outcome [13, 16]. Also in the present cohort, tumour patients were older and showed a longer follow-up compared to “all patients without malignancy” (Table 1). Hence, a tailored control group (“matched controls”) was selected accordingly from all controls. The matched controls showed consequently no statistically significant differences in “age at transplantation” and “time of observation” (Table 1). Nevertheless, some associated factors for the appearance of de-novo malignancies were detected. Patients with malignancies underwent more re-transplantations, received fewer kidneys from living donors, had a longer period of dialysis before the transplantation and had more frequently the male sex. Statistically significant differences in survival of the graft after transplantation as well as the number of mismatches between the groups were not detected (Table 1).

**Table 1:** Demographics and parameters of investigation after kidney transplantation.

	patients with malignancy	matched control without malignancies	p	all patients without malignancy	p
	n = 154	n = 154		n = 1263	
age at transplantation years (±SD)	54.5 (±13.9)	54.5 (±13.8)	0.996	49.3 (±14.8)	**<0.001**
age at tumour diagnosis* years (±SD)	60.3 (±13.3)	n/a		n/a	
donor age years (±SD)	53.4 (±15.5)	55.2 (±15.1)	0.314	52.7 (±14.7)	0.586
living donations n (%)	36 (23%)	45 (29%)	0.300	428 (34%)	**0.008**
time of observation after transplantation years (±SD)	9.8 (±5.1)	10.8 (±4.7)	0.060	8.0 (±5.3)	**<0.001**
female sex n (%)	46 (30%)	61 (40%).	0.094	507 (40%)	**0.014**
re-transplantation during follow-up n (%)	12 (8%)	4 (3%)	0.069	38 (3%)	**0.008**
dialysis before transplantation months (IQR)	49 (27–74)	32 (16–67)	**0.011**	43 (17–77)	0.212
mismatches n (±SD)	2.8 (±1.7)	2.9 (±1.7)	0.554	2.7 (±1.7)	0.658
follow-up after diagnosis of tumour^**†**^ years (±SD)	4.0 (±3.8)	5.0 (±3.6)	**0.013**	n/a	
transplant 5-year survival censored for death	88.7%	91.4%	0.448	87.6%	0.645
transplant 10- year survival censored for death	73.1%	78.5%	0.573	74.2%	0.708

* age at the first tumour diagnosis was chosen for the 23 patients with multiple malignancies.

† in matched controls: follow-up after corresponding moment in time after transplantation.

### Characteristics and distribution of all malignancies

**Fig 1:**
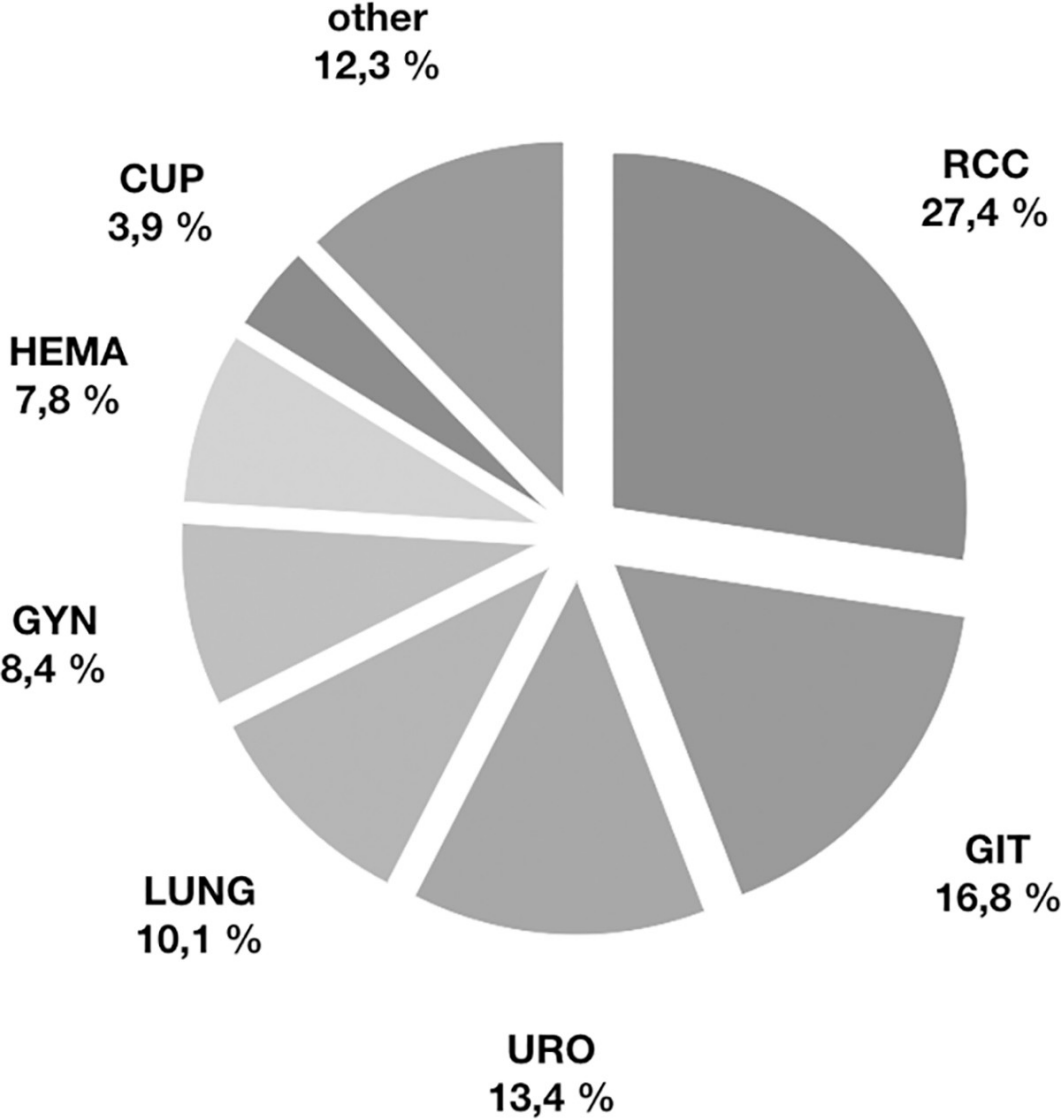
Percentage of the different tumour entities after kidney transplantation. RCC–renal cell carcinoma, GIT–tumours of the gastrointestinal tract, URO–tumours of the urogenital tract, LUNG–tumours of the pulmonary tract, GYN–gynaecology tumours, HEMA–PTLD and tumours of the blood cells, CUP–cancer of unknown primary, other–all other solid malignancies.

179 malignancies after transplantation were found in 154 patients (21 patients developed two different malignancies, in two patients three different malignancies were found). 16 of these malignancies were diagnosed after the ending of immunosuppressive therapy following terminal graft loss. In one of the 154 tumour patients cancer became clinically evident <4 weeks after KT and was probably pre-existing, in another the cancer was transmitted via the allograft organ [17]. The most frequent malignancy screened posttransplant was renal cell carcinoma (RCC, Table 2). It represented more than a fourth of all found malignancies with the highest incidence (Fig 1; Table 2). Together with the cancer of the gastrointestinal tract (GIT, summarizing malignancies of colon, pancreas, oesophagus and liver) and cancers of the urinary system (URO, summarizing prostate gland, bladder, ureter and testicle), the three most frequent tumour entities represented more than 50% of all malignancies (Fig 1; Table 2). Cancers of the lung and the bronchial tubes (LUNG) had an incidence of 1.54 per 1000 patient years (Table 2) and represented 10% of all diagnosed tumour entities (Fig 1). The malignancies of the female reproductive system characterised by vagina, uterus, ovaries and breast, were summarized as GYN and represented 8.4% of all tumours. Seven cancers of unknown primary (CUP, incidence 0.60, Table 2) were detected. Posttransplant proliferative disorders (PTLD) (n = 3) were diagnosed according to the classification of the World Health Organization (WHO) and summarized in HEMA, together with other haematological tumours [18, 19]. The tumour entity “other” represents all other malignancies, mainly cancer of the skin (malignant melanoma), Kaposi´s sarcoma and malignancies of head and neck (larynx, tonsils, pharynx and thyroid gland) (Fig 1; Table 2).

**Table 2:** Characteristics at diagnosis of malignant tumours after kidney transplantation.

tumour entity	n	time of diagnosis after Tx years (±SD)	age at diagnosis years (IQR)	female sex n	mortality due to tumour entity n (%)	incidence ^†^
**RCC**	49	6.7 (±4.9)	61 (48–69)	6 (12%)	6 (12%)	4.20
**GIT**	30	5.7 (±3.8)	69 (62–73)	7 (23%)	15 (50%)	2.57
**URO**	24	5.2 (±3.1)	66 (60–71)	2 (8%)	3 (13%)	2.06
**LUNG**	18	5.6 (4.6)	69 (64–75)	5 (28%)	16 (89%)	1.54
**GYN**	15	6.6 (±4.2)	44 (36–55)	14 (93%)	2 (13%)	1.29
**HEMA**	14	5.1 (±3.1)	57 (48–71)	6 (43%)	3 (21%)	1.20
**CUP**	7	9.5 (±4.9)	69 (63–74)	4 (57%)	7 (100%)	0.60
**other**	22	6.5 (±5.0)	64 (47–70)	8 (36%)	6 (27%)	1.89
**all**	179	6.2 (±4.3)	65 (52–71)	52 (29%)	58 (32%)	15.34

RCC–renal cell carcinoma, GIT–tumours of the gastrointestinal tract, URO–tumours of the urogenital tract, LUNG–tumours of the pulmonary tract, GYN–gynaecology tumours, HEMA–PTLD and tumours of the blood cells, CUP–cancer of unknown primary, other–all other solid malignancies

† calculated per 1000 patient years.

RCC mainly affected males and was characterised by the lowest mortality rate, likewise, URO rarely affected females and showed low rates of cancer specific death (Table 2). Compared to other tumours and specifically to RCC and GIT, URO appeared early after transplantation (Table 2). A different outcome was seen after diagnosis of GIT. Both the percentage of affected woman and the mortality were elevated compared to RCC and URO (Table 2). GYN developed in younger patients and also affected one man (Table 2) [17]. Tumour related mortality was particularly high in cases of lung cancer and CUP (Table 2). Haematological malignancies as well as PTLD (summarized as HEMA) appeared comparable early posttransplant and were similarly distributed between females and males (Table 2).

### Incidence of the first malignancy

**Fig 2:**
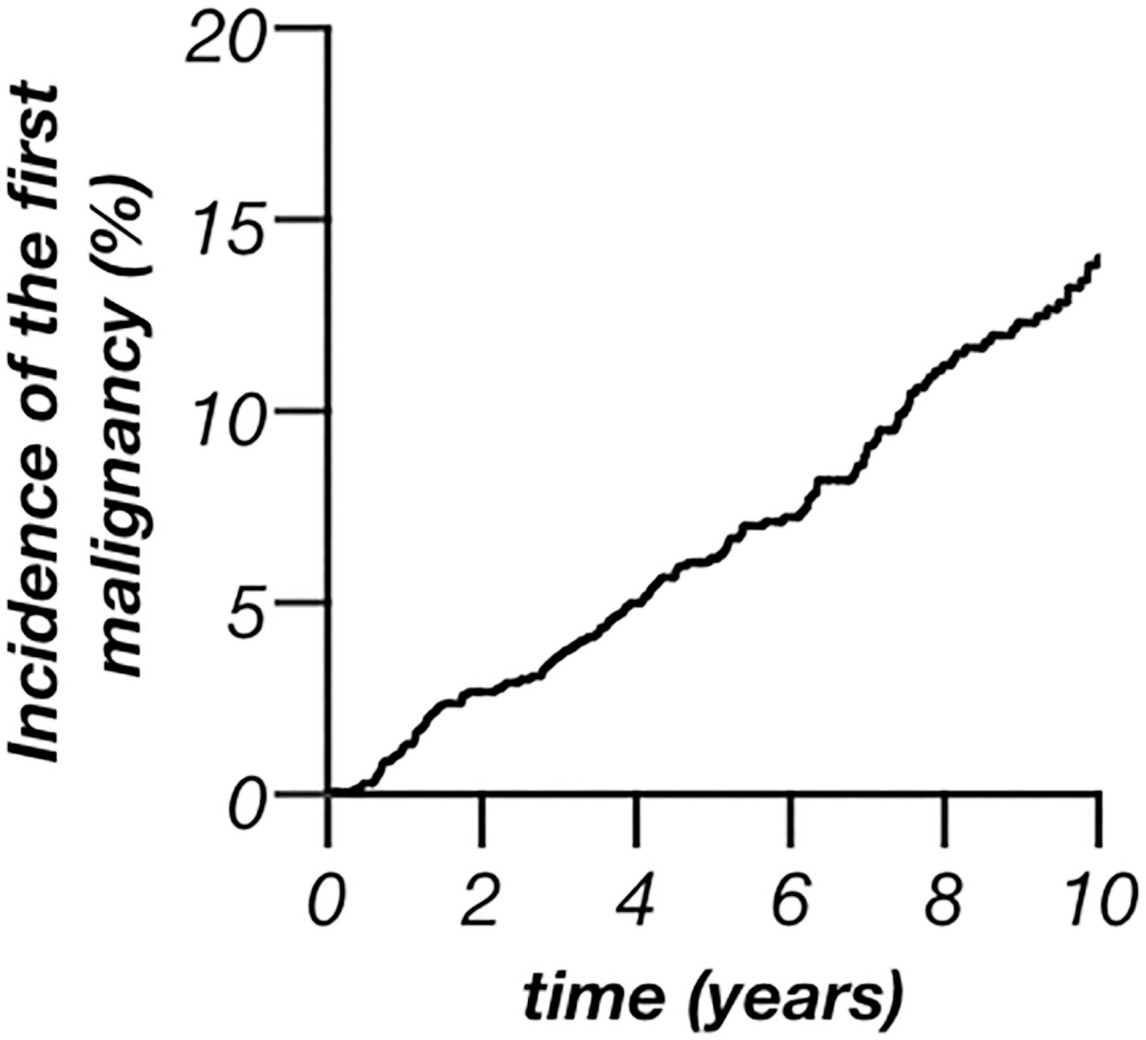
Incidence of the first malignancy after the first kidney transplantation. Incidence of the first malignant tumour for the first ten years after kidney transplantation.

The incidence of the first malignancy (n = 154) after transplantation amounted to a total of 15.34 per 1000 patient years and is displayed for the first 10 years after KT in Fig 2. A predicted tumour incidence for the first malignancy of 1.2% (CI 0.6–1.8%; males: 1.7%, CI 0.9–2.5%; females: 0.6%, CI 0.0–1.2%) after one year was calculated, that increased to 6.2% (CI 4.8–7.5%; males: 7.1%, CI 5.3–8.9%; females: 4.7%, CI 2.7–6.7%) after five and 14.0% (CI 11.6–16.6%; males 15.8%, CI: 12.7–18.9%; females 11.1%, CI 7.6–14.6%) after ten years, respectively (Fig 2; sex-specific data are shown in S1 Fig).

### Survival after transplantation, after diagnosis of the first malignancy and for specific tumour entities

**Fig 3:**
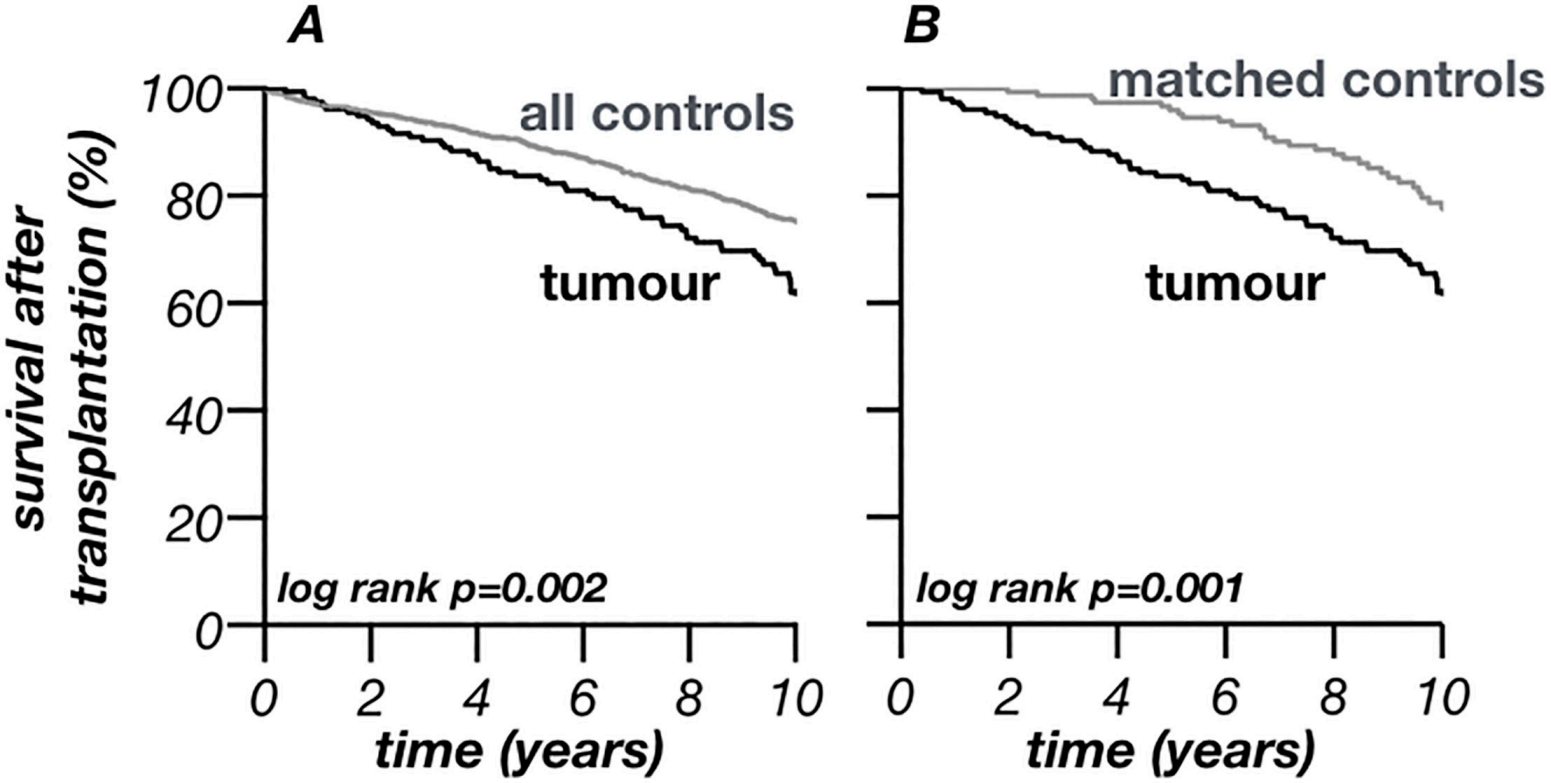
Survival after transplantation. Panel A–Transplanted patients with malignancy versus all controls (all transplanted patients without malignancy). Panel B–Transplanted patients with malignancy versus cancer free matched-controls.

The survival after KT was calculated for all included patients and is shown for patients with malignancy (n_tumour-group_ = 154) versus all patients without malignancies (n_all-controls_ = 1263) (Fig 3 –panel a) and matched controls (n_match_ = 154) (Fig 3 –panel b). The calculated mortality of the tumour-group after one year was 2.6% (CI 0.1–5.1%) compared to 3.0% (CI 2.1–4.0%, all controls) and 0.0% (matched controls). The survival decreased over time to 83.6% (CI 77.8–89.5%) after five and 62.0% (CI 53.7–70.2%) after ten years in the tumour-group. Controls showed lower mortalities five (all controls: 10.6%, CI 8.8–12.5%; matched controls: 4.0%, CI: 0.9–7.2%) and ten years after KT (all controls: 24.8%, CI 21.8–27.8%; matched controls: 22.3%, CI: 14.9–29.7%) to a statistically significant degree (Fig 3).

**Fig 4:**
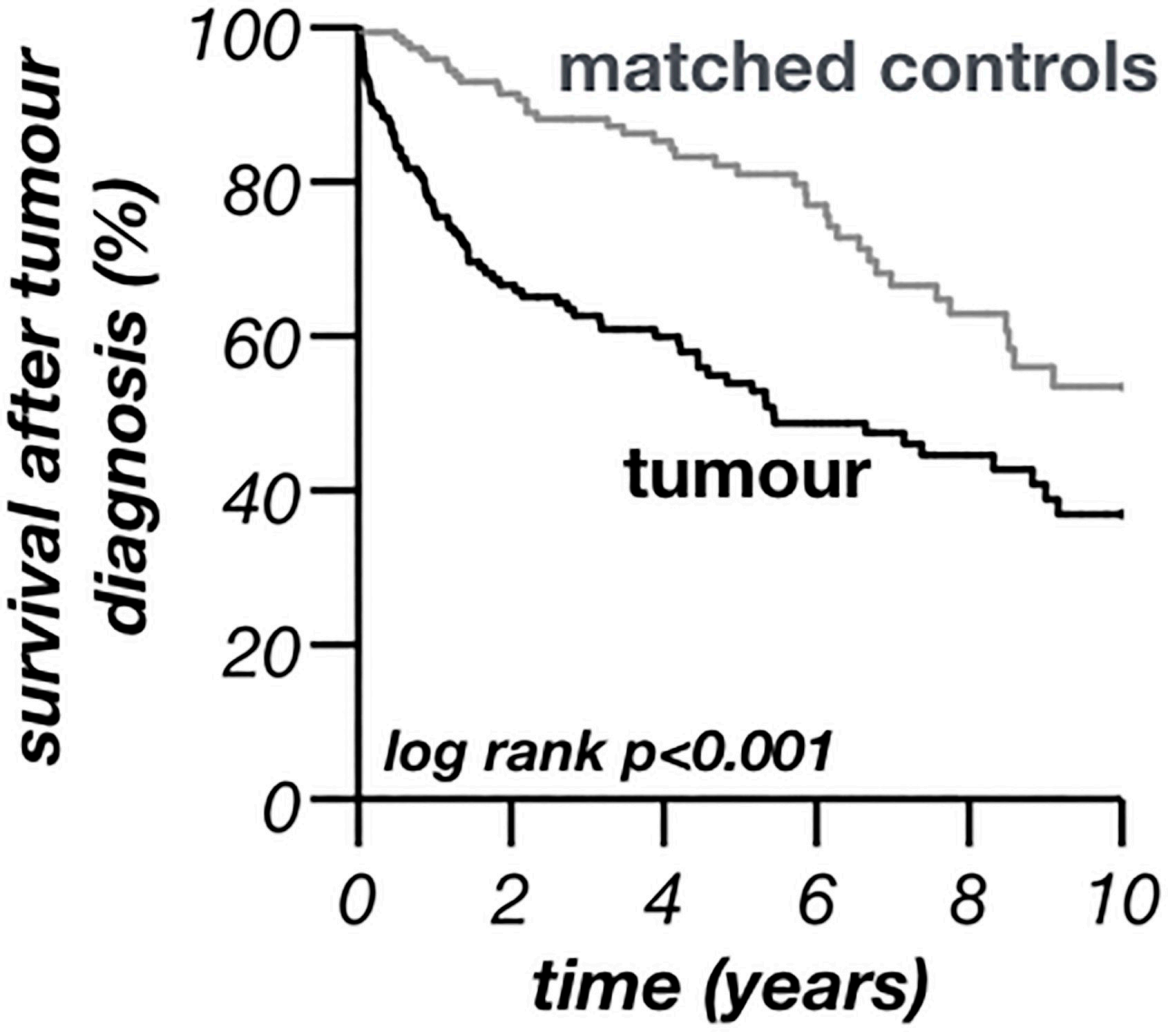
Survival after diagnosis of a malignant tumour. Survival after diagnosis of a malignant tumour of the cancer free matched controls was calculated accordingly to the period after transplantation of the assigned tumour patient.

Besides the survival after KT, the survival of the tumour-group after diagnosis of the first malignancy was calculated. The correspondent period after transplantation was followed for each of the cancer-free matched controls. The analysis reveals a high mortality within the first years after tumour diagnosis (mortality after one year 23.9%, CI: 17.1–30.7%) compared to matched controls (4.1%, CI: 1.0–7.2%). The difference in survival is statistically highly significant (p<0.001) and mortality remains elevated in the tumour-group after five (46.1%, CI: 37.4–54.8% versus 19.0%, CI 11.9–26.2%) and ten years (63.1%, CI: 52.8–73.4% versus 46.6%, CI: 34.1–59.1%) (Fig 4).

**Fig 5:**
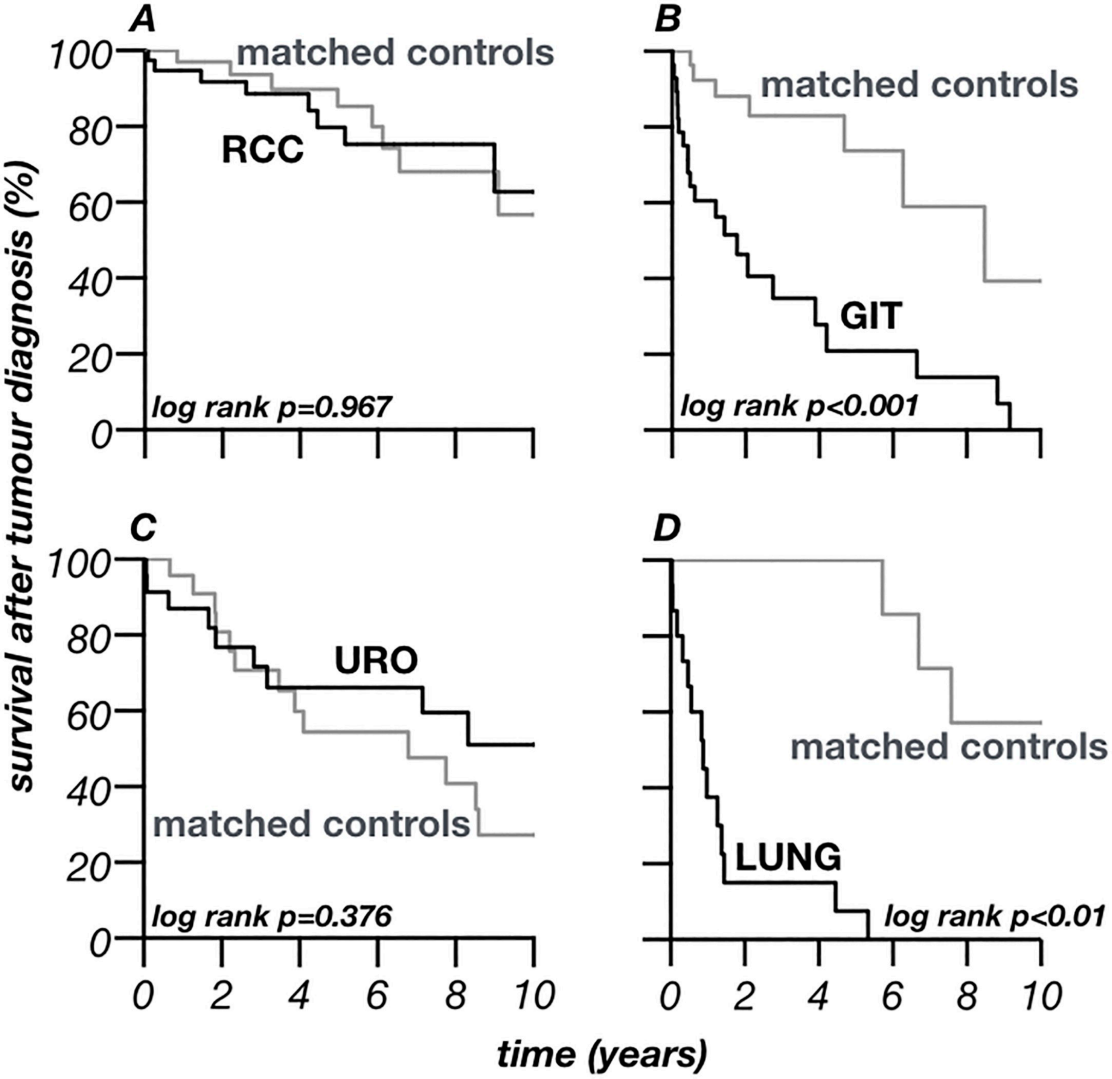
Survival after diagnosis of specific carcinomas. Panel A—Survival after diagnosis of renal cell carcinoma. Panel B—Survival after diagnosis of cancer of the gastrointestinal tract. Panel C—Survival after diagnosis of cancer of the urinary system. Panel D—Survival after diagnosis of cancer of the respiratory system. Survival after diagnosis of a malignant tumour of the cancer free matched controls was calculated accordingly to the period after transplantation of the assigned tumour patient.

Patients´ survival after diagnosis of a malignant cancer was also calculated for the different tumour entities and is summarized in Table 3. The estimated 1-year survival after diagnosis of RCC was high and remained high over time (Table 3) with only little differences in survival compared to matched controls (Fig 5 –panel a). The estimated survival within the first years after diagnosis of cancers of the female reproductive system was even higher and decreased slightly after five years (Table 3). Also the estimated survival after diagnosis of cancers of the urinary system was high and did not differ to a statistically significant degree from matched controls (Table 3; Fig 5 –panel c). Life expectancy after diagnosis of a tumour of the respiratory system was low and showed a statistically significant difference to matched controls, similar results were seen for CUP and GIT (Table 3; Fig 5 –panels b and d; S1 Table).

**Table 3:** Estimated survival after the diagnosis of the first malignancy.

tumour entity	patients with first malignancy	1-year survival	3-year survival	5-year survival
**RCC**	38	95%	89%	80%
**GIT**	28	61%	35%	21%
**URO**	23	87%	72%	66%
**LUNG**	15	38%	15%	8%
**GYN**	12	100%	100%	80%
**HEMA**	13	92%	85%	75%
**CUP**	6	17%	0%	0%
**other**	19	71%	54%	54%
**all**	154	76%	63%	54%
**matched controls**	154	96%	88%	81%

RCC–renal cell carcinoma, GIT–tumours of the gastrointestinal tract, URO–tumours of the urogenital tract, LUNG–tumours of the pulmonary tract, GYN–gynaecology tumours, HEMA–PTLD and tumours of the blood cells, CUP–cancer of unknown primary, other–all other solid malignancies.

## Discussion

### Risk factors for the development of cancer and survival after transplantation

With the improved long-term outcomes after kidney transplantation, the incidence of de-novo malignancies after transplantation has risen [13]. The present investigation systematically screened for tumours in all patients who underwent the first kidney transplantation between 01.01.1995 and 31.12.2016 at our centre. Epidemiological data such as age at transplantation, age at tumour diagnosis and cancer incidence is in line with data from Europe (Table 1) [10, 20]. Compared to the general population transplanted people are young at diagnosis of cancer [13, 21–23]. The present investigation underpins this finding. The present cohort was younger at tumour diagnosis (65 years [IQR 52–71], Table 2) compared to national analysis of the German tumour population (mean age of 69–70 years) [24]. The younger age at tumour diagnosis even persists after excluding RCC from the present analysis (mean age at tumour diagnosis without RCC: 66.1±13.5 years) that is well known to be associated with ESRD in younger transplant recipients. The mean age at first tumour diagnosis was even much younger, both in all 154 transplanted patients with malignancy (60.3±13.3 years [Table 1]) and in the 116 patients whose first tumour was not RCC (mean age at tumour diagnosis after excluding RCC as first tumour: 61.3±13.6 years). An earlier investigation found an association between deceased kidney donation and recipients´ age at transplantation [25]. This was also seen in the present investigation. However, matched-controls received more likely a living donation (29%) than patients with malignancy (23%). Although this difference was not statistically significant it points towards a potential association between de-novo malignancies and deceased donor transplantation. Also other earlier associations as male sex, numbers of re-transplantations and time on dialysis before KT were seen in the present study (Table 1) [10, 13, 16, 21, 23, 26, 27]. Nevertheless, it was hypothesized that time on dialysis might trigger pre-existing cancers rather than de-novo malignancies [2]. The fact that the kidney transplantation itself is a risk factor for malignancies is furthermore underpinned by the moment of a tumour´s development: a steep increase of the incidence of the first malignancy was seen within the first two years after transplantation (1.2% per year). Later incidence still increases constantly but levels off over time (Fig 2). Yet, it remains elevated compared to the general German population (0.35% in women, 0.43% in men, respectively; data from 2013) [24]. The cancer incidence of the present investigation is in line with previous single-centre studies as well as data from the Australia and New Zealand Dialysis and Transplant Registry although higher compared to data from US registries [13, 28–30]. 44.6% malignancies were found five, 81.0% malignancies ten years posttransplant, respectively. This is in line with earlier investigations from Hungary (20% within the first year) and the greater Munich area (45% five, 71% ten years after KT) [13, 16]. Earlier studies explained this phenomena with the theory of dormant malignancies that are antedated and accelerated by the immunosuppression after KT [13]. This theory consequently leads to a lower mean of age at diagnosis and helps to explain the association between the time of observation and incidence of malignancies (Table 1) [2, 13, 16, 20, 31]. Moreover, malignancies after transplantation seem to correlate with a higher mortality, an excess risk of cancer-related death of over 2.5 times was shown after KT [2]. The present data also showed significantly worse survival in the tumour-group, both after KT (Fig 3) and after tumour diagnosis (Fig 4). Similar to earlier investigations, mortality after tumour diagnosis was elevated [20, 32]. However, the differences to matched controls were mainly seen within the first years after diagnosis and mortality rates became similar later (Table 3; Fig 4). Moreover, after a survival of around four years with malignancy the further expectancy of life was hardly altered compared to kidney-transplanted patients without tumour. Cancer specific mortality varied by age, sex, year of transplantation and type of cancer. Most of the patients with cancer specific cause of death died within these first four years after diagnosis [2]. Interestingly, the 5- and 10-year survival of the transplant did not differ significantly between the groups (Table 1).

### Specific cancers and their epidemiological analyses

The distribution and frequency of cancer types varies by the transplanted organ and differs to the general population [2, 16]. The high prevalence of renal cell carcinoma (RCC), malignancies of the gastro-intestinal tract (GIT) and malignancies of the urinary system (URO) (Fig 1; Table 2) were also seen in earlier investigations, both in single-centre observations and national registries [20, 33, 34]. In Germany in 2014 RCC represented with 2.4% of all new cancers in females (3.8% in males) the 11^th^ most common tumour entity (6^th^ most common entity in males, respectively); incidence was specified with 13.3 in females (23.9 in males, respectively) per 100´000 patient years [24]. It was already shown that RCC is specifically increased after KT and even approximately 4,9–15 fold more common compared to the general population and other organ recipients [16, 29, 31, 34–36]. The malignant transformation of kidney cysts was assumed as one possible explanation [20, 37].

According to the literature mortality of RCC was low in the present investigation, even the lowest among all investigated malignancies (Table 2) [2, 38]. Interestingly, the survival after diagnosis of RCC seems to be similar after KT (80% after five years; Table 3) compared to the data of the national registry (77%) [24]. This is in line with data after KT from the Frankfurt Transplant Center (83.5%) and Portugal (91.3%) [29, 38]. The good outcome of RCC after KT might be caused by early detection of smaller cancer sizes, asymptomatic malignancies, low-stage and low-grade tumours due to the intensive monitoring of patients with end stage renal diseases posttransplant [20, 23].

Cancers of the gastro-intestinal tract are common after SOT and in the general population [16, 24, 36, 39]. In Germany, almost every 8^th^ cancer affects the colon. Colorectal cancer is even the second most frequent tumour entity in women (third in men) with a proportion of 26% of all carcinomas (33% in man, respectively). The incidence for Germany was calculated with 67.6 per 100´000 patient years for women (83.4 for man) [24]. Rocha et al. counted colorectal cancer even as the most frequent non-cutaneous tumour after KT, in contrast to a Swiss investigation that did not find a statistically significant increase [20, 39]. Also in the present investigation cancers of the bowel (mainly colorectal cancer) represented a big proportion of the GIT category (17/30; S1 Table). Cancers of pancreas, oesophagus and liver (that were summarized as GIT in the present investigation) are common entities, too [24]. Age at and time of diagnosis of GIT after KT are in line with earlier publications [40]. Survival after colorectal cancer depends significantly on the stage of disease but is generally poor after SOT and worse compared to the general population [5, 40–42]. This fact is underlined by the present investigation. Mortality is elevated to matched controls (Tables 2 and 3, Fig 5 - panel B). The 5-year survival of 21% in the GIT category is compared to the general German population rather on the level of pancreas carcinoma (8–9%) than colon carcinoma (51–52%) [24]. The poor outcome of the GIT category is not a consequence of over-representation of tumours known to be associated with worse prognosis. Mortality is high for all cancers, except stomach in this heterogeneous group (S1 Table).

Malignancies of the urinary system are increased after KT, but the increase of tumours of the prostate gland (PC; n = 15) was less pronounced in the present investigation as described earlier [20, 29, 33, 38]. Two cancers of the bladder (ICD-10: C67; n = 6) were seen in females while all other malignancies of the urinary system were seen in man (Table 2). However, the true incidence of PC is difficult to calculate because the number depends on whether systematic screening (e.g. PSA, digital rectal examination) was performed or not. Independent of the incidence, PC related death is rare [32, 38]. No one died as a direct consequence of PC in the present investigation and survival was similar to matched controls (Fig 5 - panel c). Nevertheless, the estimated 5-year survival rate after tumour diagnosis was reduced (66%, Table 3) compared to data of the general German population (76%), while incidence was elevated (2.05 [Table 2] versus 1.52 per 1´000 patient-years). Also the age at diagnosis is less in the present cohort (66 years [IQR 60–71], Table 2) compared to national data (71–72 years) [24]. As PC mainly affects older men, these patients probably died from competing risks after KT compared to non-transplanted patients. Moreover, among urological tumours in patients after KT, a higher rate of bladder cancers have been described earlier also for Germany [29]. Cancer of the bladder is decreasing in frequency in the German population but remains a cancer of male patients (about 75%) [24].

Tumours of the lungs and bronchial tubes are frequent malignancies after SOT, especially in lung recipients but also after KT [5, 13, 20, 30, 37]. Moreover, it is common in the general population and the second (males, 13,9%) and third (females, 8,5%) most common malignancy in Germany [13, 24]. Even if the proportion seems to be similar in the German population and in the present investigation, the incidence is not. The present investigation is in line with international data after KT and elevated compared to data of the German tumour registry (46.6 [females] and 89.5 [males] per 100´000 patient-years [24, 37]. The survival after this diagnosis is poor, both after KT (Table 3; Fig 5 - panel d) and in the general population (5 year: 15–20%) [20, 24, 32]. Tobacco is the main risk factor, also after KT. Earlier investigations found an increased risk for lung cancer after KT, especially in females, that was not seen in the present study (Table 2) [10].

Cancers of the female reproductive system are sex-specific and mainly represented breast cancers (Table 2). In the present investigation one male received breast cancer via the allograft organ [17]. This is fortunately a very rare case [9]. With regard to German data (age at diagnosis of breast cancer: 64 years), the present cohort was younger at tumour diagnosis (44 year [IQR 36–55], Table 2) and might not be included in the national mammography screening program (50–69 years). Interestingly, the 5-year survival after tumour diagnosis is comparable between the present cohort and German data (79–88%). [24].

Non-Hodgkin Lymphomas, as B-cell Lymphoma and PTLD, are common tumours after SOT [30, 37]. The incidence of PTLD varies between the authors and countries and correlates with a comparable high mortality similar to the present data (Table 2) [30, 43, 44].

Strengths of the present investigation are the complete and long follow-up after KT in one single centre with the entire medical history of every kidney-transplanted patient including data of the local cancer registry. As a consequence one limitation is the reduced number of patients. Another limitation is the data on immunosuppression. Switching immunosuppressant doses and drugs is nowadays a common practice, which makes the precise description of immunosuppressive load rather complex [45]. Even if the exact IS regime of each patient could not be considered, this potential confounder was reduced by the matching procedure, which accounted for time under immunosuppression, era of transplantation as well as patient´s age and sex. Non-melanoma skin cancer (ICD-10: C44), a frequent cancer after SOT, was not integrated in the present investigation as the dermatological follow-up is performed in external departments and reliable complete data was not available [26, 36, 46].

## Conclusion

Incidence and mortality are increased and antedated under immunosuppression but the duration of dialysis seems to be associated with cancer, too [13, 47–49]. Although we cannot tell how many cancers were recognised by cancer screening, early (and asymptomatic) detection of malignancies might help to optimize long-term results after transplantation. Therefore, the present investigation not only reinforces earlier postulations to perform national screening programs more rigorously in kidney transplant recipients. With regard to present results, previous calls for a more tailored cancer screening after KT are strengthened [40, 50]. This can include low dose CT scans of the chest in case of previous lung diseases or present or history of smoking as well as more frequent colonoscopies and occult blood tests in renal transplant recipients [51–54]. Our centre performs annual abdominal ultrasounds including the native kidneys in order to detect RCCs at early stages. As kidney transplant recipients are younger at tumour diagnosis, the expansion of national screening programs (e.g. mammography, colonoscopy, low dose chest CT) for this population should be discussed and investigated [54]. However, frequent co-morbidities are competing risks, which might reduce the benefit of standardized national cancer screening programs [9]. Consequently, further research is needed to develop rationally tailored tumour screening programs for renal allograft recipients.

## Supporting information

S1 Fig Sex-specific incidence of the first malignancy after the first kidney transplantation. Incidence of the first malignant tumour for the first ten years after kidney transplantation for females (grey) and males (black).
(TIF)

S1 Table Distribution and mortality of the different tumour entities of the GIT category. (DOCX)
