# Supplementary figures and images for: De-novo malignancies after kidney transplantation: A long-term observational study
Source: PLoS One. 2020 Nov 30;15(11):e0242805. doi: 10.1371/journal.pone.0242805 (PMC7703884; doi:10.1371/journal.pone.0242805)

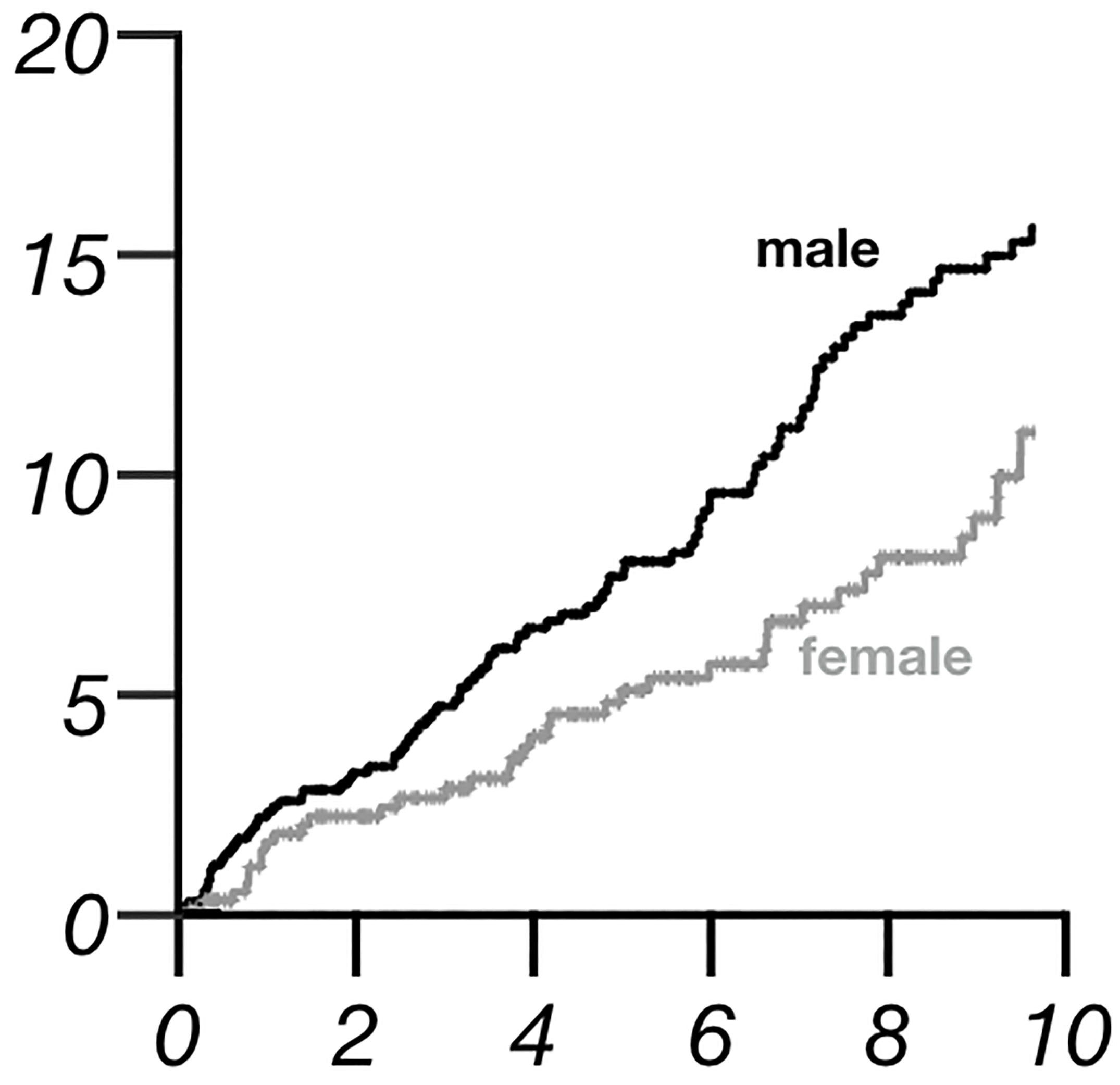

Supplement: S1 Fig — Incidence of the first malignant tumour for the first ten years after kidney transplantation for females (grey) and males (black). (TIF) [file pone.0242805.s001.tif]
