# Supplementary material for: De-novo malignancies after kidney transplantation: A long-term observational study
Source: PLoS One. 2020 Nov 30;15(11):e0242805. doi: 10.1371/journal.pone.0242805 (PMC7703884; doi:10.1371/journal.pone.0242805)
Supplement: S1 Table — (DOCX) [file pone.0242805.s002.docx]

**S1 Table. Distribution and Mortality of the different tumour entities of the GIT category.**

| **Cancer Type** | **ICD-10 Code** | **Included cases** | **Mortality due to tumour, n (%)** |
| --- | --- | --- | --- |
| Cancer of the bowel | C18 – C22 | 17 | 7 (41.2%) |
| Cancer of liver and gallbladder | C22 – C24 | 6 | 5 (83.3%) |
| Oesophageal Cancer | C15 | 3 | 1 (33.3%) |
| Pancreatic Cancer | C25 | 2 | 2 (100%) |
| Cancer of the stomach | C16 | 2 | 0 (0.0%) |
| **All** | **C15 – C24** | **30** | **15 (50.0%)** |

* Cancer of the bowel is mainly characterised by colorectal cancer (n=15)
